# Leveraging a Cloud-Based Critical Care Registry for COVID-19 Pandemic Surveillance and Research in Low- and Middle-Income Countries

**DOI:** 10.2196/21939

**Published:** 2020-11-23

**Authors:** CRIT Care Asia, Madiha Hashmi, Abi Beane, Srinivas Murthy, Arjen M Dondorp, Rashan Haniffa

**Affiliations:** 1 Collaboration for Research, Improvement and Training in Critical Care in Asia Mahidol Oxford Tropical Medicine Research Unit Faculty of Tropical Medicine, Mahidol University Bangkok Thailand; 2 Please see acknowledgements section for list of collaborators Bangkok Thailand; 3 Department of Critical Care Ziauddin University Karachi Pakistan; 4 Department of Pediatrics University of British Colombia Vancouver, BC Canada; 5 Collaboration for Research, Improvement and Training in Critical Care in Asia Mahidol Oxford Tropical Medicine Research Unit Bangkok Thailand

**Keywords:** critical care, registry, informatics, COVID-19, severe acute respiratory infection, pandemic, surveillance, cloud-based, research, low-and-middle-income countries

## Abstract

The COVID-19 pandemic has revealed limitations in real-time surveillance needed for responsive health care action in low- and middle-income countries (LMICs). The Pakistan Registry for Intensive CarE (PRICE) was adapted to enable International Severe Acute Respiratory and emerging Infections Consortium (ISARIC)–compliant real-time reporting of severe acute respiratory infection (SARI). The cloud-based common data model and standardized nomenclature of the registry platform ensure interoperability of data and reporting between regional and global stakeholders. Inbuilt analytics enable stakeholders to visualize individual and aggregate epidemiological, clinical, and operational data in real time. The PRICE system operates in 5 of 7 administrative regions of Pakistan. The same platform supports acute and critical care registries in eleven countries in South Asia and sub-Saharan Africa. ISARIC-compliant SARI reporting was successfully implemented by leveraging the existing PRICE infrastructure in all 49 member intensive care units (ICUs), enabling clinicians, operational leads, and established stakeholders with responsibilities for coordinating the pandemic response to access real-time information on suspected and confirmed COVID-19 cases (N=592 as of May 2020) via secure registry portals. ICU occupancy rates, use of ICU resources, mechanical ventilation, renal replacement therapy, and ICU outcomes were reported through registry dashboards. This information has facilitated coordination of critical care resources, health care worker training, and discussions on treatment strategies. The PRICE network is now being recruited to international multicenter clinical trials regarding COVID-19 management, leveraging the registry platform. Systematic and standardized reporting of SARI is feasible in LMICs. Existing registry platforms can be adapted for pandemic research, surveillance, and resource planning.

## Introduction

The COVID-19 pandemic has revealed limitations in capacity for real-time surveillance needed for responsive health care action in low- and middle-income countries (LMICs), where infrastructure and institutional partnerships to facilitate accurate and timely reporting of clinical and operational data are often absent [1,2]. This absence of data impedes the ability of researchers, clinicians, and health policy leaders to identify context-specific risk factors associated with severe disease or death and make informed decisions regarding public health policy, critical care admission, and management of patients with severe acute respiratory infection (SARI) [3,4].

Critical care services are a key component of pandemic preparedness in health systems [5]. In LMICs, critical care services are already limited outside the pandemic context, and technical and human resources are often already overburdened with existing endemic illness [6]. Limitations in resources, diagnostics, and training are potential barriers to the operationalization of internationally comparable surveillance and translational research in many LMICs. The paucity of LMIC representation in international datasets and in research risks disenfranchising large parts of the world [7].

The effectiveness of a response to a pandemic threat depends critically on the speed and focus of that response. At the core of the World Health Organization (WHO) plan is the Clinical Characterization Protocol (CCP) developed by the International Severe Acute Respiratory and emerging Infections Consortium (ISARIC) [8]. ISARIC aims to facilitate real-time research on diseases caused by novel pathogens of public health concern to save lives and inform public health policy early in and during outbreaks [8]. The open-access protocols use standardized and refined case report forms, information documents, and consent documents, and they offer a tiered (0-3) biological sampling schedule. The WHO Ethics Review Committee approved a global master protocol for the ISARIC CCP and endorsed its use in outbreaks of public health interest [9].

Pakistan Registry of Intensive CarE (PRICE) was established in 2018 with the support of Wellcome. PRICE supports a national network of 49 sites in Pakistan recording over 2000 monthly critical care admissions [10]. PRICE provides real-time reporting on the epidemiology, severity of illness, treatment, microbiology, and outcomes of intensive care unit (ICU) patients alongside information regarding the workforce, unit activity, unit acuity, and resource utilization in the ICU. Work already undertaken by PRICE has identified wide regional disparity in the availability of critical care resources [11]. Similarly, characterization of the current pandemic in relation to available resources is an essential tool in the management of an outbreak [6]. The PRICE platform, codesigned with clinicians, leverages the NICS-MORU platform, a cloud-based system that allows real-time monitoring of case mix, performance metrics, and benchmarking for acute and critical care; it is currently deployed in over 11 LMICs and 180 acute and critical care units in South Asia and sub-Saharan Africa [3]. PRICE and NICS-MORU are founding members of the Wellcome-MORU Collaboration for Research, Improvement and Training in Critical Care Asia (CRIT Care Asia), established in August 2019, which will support adaptation and implementation of the PRICE reporting model in nine countries in South and Southeast Asia: Bangladesh, India, Laos, Nepal, Malaysia, Pakistan, Sri Lanka, Thailand, and Vietnam.

This viewpoint describes the adaptation and operationalization of the PRICE platform to conform with all tiers of the ISARIC CCP for SARI in participating ICUs. It also outlines the development of a rapidly deployable standalone SARI application for use in ICUs and acute care facilities in Pakistan and beyond.

## Approach

The ISARIC tier 0-3 CCP was incorporated into the PRICE platform in March 2020. COVID-19 diagnosis was mapped to existing Acute Physiology and Chronic Health Evaluation (APACHE) IV diagnostic codes [12]. Variables were added using a standardized nomenclature, Systematized Nomenclature of Medicine Clinical Terms (SNOMED CT), that was already operationalized in the platform, enhancing interoperability at the organizational level and facilitating sharing with ISARIC [13].

**Figure 1:**
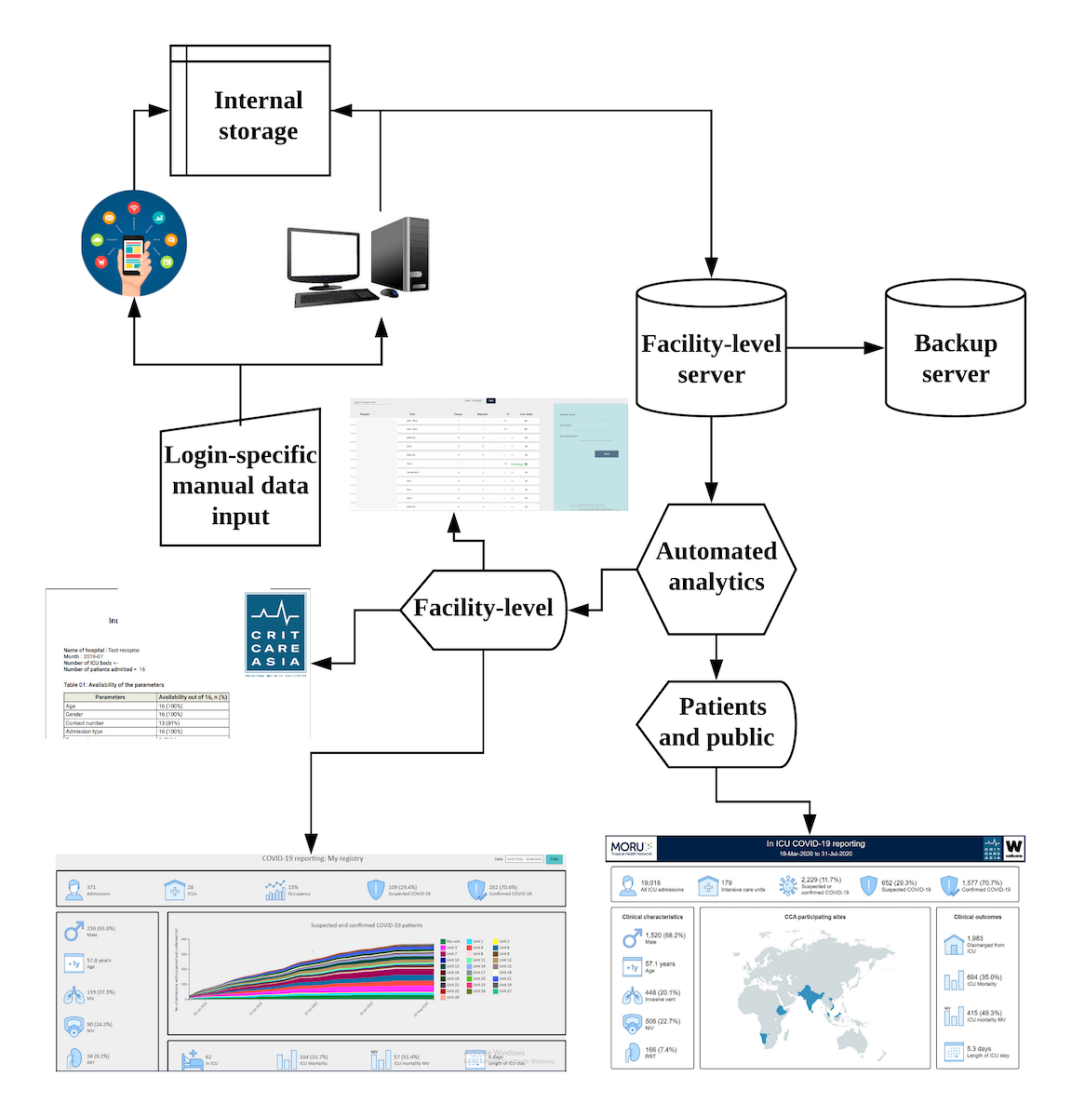
Scheme outlining the process of data entry, storage, and visualization in the Pakistan Registry for Intensive CarE (PRICE) platform.

Leveraging the existing PRICE data collection methods and the ISARIC CCP protocol, training was provided to existing data collectors and clinical leads in the additional data set and in dashboard navigation. The process of data entry, storage, and visualization is illustrated in Figure 1. Training was facilitated by the Wellcome-funded NICS-MORU registry development and support team based in Sri Lanka. The NICS-MORU team facilitates registry implementation and adoption across sites in South Asia and sub-Saharan Africa. The team provided remote training via web-based videoconferencing and instant messaging apps, overcoming challenges such as restricted travel. These processes, along with other implementation and data monitoring tools, have been explained in detail in previous publications [3].

In addition, 24-hour support via messaging provided operational, data entry, and technical assistance. Inbuilt platform features (completeness, validation, consistency) and raw data capture ensure the quality of the data for surveillance and subsequent research [14]. Platform-enabled reports and dashboards visualize real-time operational, clinical, and outcome data of suspected and confirmed COVID-19 cases in the ICUs. Metrics including case mix, service utilization, and unit performance are made accessible to the relevant stakeholders.

A web-based report and dashboard were integrated within the existing PRICE platform to visualize real-time operational, clinical, and outcome data of suspected and confirmed COVID-19 cases in the ICUs. Metrics included use of respiratory support, renal replacement therapy and outcomes, and the availability of ICU beds and ventilators. Dashboards were made accessible to the relevant stakeholders through their existing logins via tablet or desktop. Printable weekly reports provide more detailed analyses of the case mix, severity of illness, microbiology, and outcomes.

The ISARIC CCP–compliant SARI reporting tool was then developed as a standalone mobile and desktop application for use in health care facilities that are not currently part of an existing registry network. Mirroring the PRICE platform, the application requires minimal data connectivity (3G) and has offline functionality. This standalone application provides a mechanism for capturing SARI case reports in a systematic and internationally comparable manner, providing rapid onboarding of health care organizations with minimal information technology infrastructure while enabling institutions to retain ownership of data and use for local service evaluation.

## Relevant Changes

ISARIC-compliant SARI reporting was successfully developed and implemented in 49 ICUs within the PRICE network over a four-week period. Data completeness for ISARIC tiers 0 and 1 was above 97%, overcoming perennial LMIC data quality challenges and ensuring that the data are suitable for high-quality research [14]. As of May 24, 2020, clinicians and health service organizers had accessed real-time information on 592 suspected and confirmed COVID-19 cases via secure portals within their ICUs. Real-time aggregate data on ICU occupancy, acuity, resource utilization, and remaining capacity is being used by ICU operational leads to better inform organizations of interdepartmental resources and coordination of regional public health strategies in partnership with members of the government’s pandemic task force. Information regarding treatment utilization (eg, ventilation, noninvasive ventilation, renal replacement therapy, vasopressors) is being used by the Faculty of Critical Care Medicine of the College of Physicians and Surgeons Pakistan to guide regional training priorities for health care workers who are being upskilled in the management and care of critically ill patients with acute respiratory illness. The same information has guided biweekly interprofessional web-based meetings with PRICE and CRIT Care Asia members discussing context-specific treatment strategies, and it has informed priorities for the management of patients with COVID-19 in LMICs [5]. PRICE-collaborating ICUs are now being recruited to registry-embedded international multicenter research clinical trials—site recruitment to the Randomized, Embedded, Multifactorial Adaptive Platform Trial for Community-Acquired Pneumonia (REMAP-CAP) COVID-19 trial is underway in three countries in the CRIT Care Asia network—with registry-enabled clinical and epidemiological data informing the selection of context-specific interventions and site enrollment. The ISARIC CCP–compliant data set and inbuilt registry audit and feedback mechanisms have since been made available to 180 ICUs within the CRIT Care Asia network. The standalone SARI reporting application is open access for future collaborators through ISARIC [8]. SARI reporting for CRIT Care Asia is also publicly available [15].

A key challenge facing CRIT Care Asia and similar international consortiums operationalizing pandemic surveillance reporting are the administrative and technical barriers to data curation and sharing. CRIT Care Asia’s federated approach to data storage, which enables sites to retain ownership of data while contributing metadata to national and international networks with minimal site-level data transformation, has helped overcome these barriers.

## Conclusion

The COVID-19 pandemic represents a major challenge to health care services worldwide, particularly for ICUs. In LMICs, the surge of SARI patients is placing unprecedented stress on existing services, infrastructure, and health care workers. For health care systems worldwide, the challenge of operationalizing disease-specific data capture during a pandemic may best be met by harnessing existing digital health solutions, such as registries, which in a nonpandemic context enable multicenter monitoring and reporting of critical care case mix, workload, and availability of ICU resources. To realize the potential of registries, however, investment is needed in robust health care technology that is capable of rapid transformation, scalability, and interoperability [2]. Those responsible for commissioning and developing registries should be mindful of the potential uses of such systems as part of a wider public health strategy and of the need to build systems with the capabilities described above. Realizing this capability in LMICs would be a significant step forward in achieving an effective and coordinated global pandemic response.

## Abbreviations

APACHE: Acute Physiology and Chronic Health Evaluation
CCP: Clinical Characterization Protocol
CRIT Care Asia: Collaboration for Research, Improvement and Training in Critical Care Asia
ICU: intensive care unit
ISARIC: International Severe Acute Respiratory and emerging Infections Consortium
LMICs: low- and middle-income countries
PRICE: Pakistan Registry for Intensive CarE
REMAP-CAP: Randomized, Embedded, Multifactorial Adaptive Platform Trial for Community-Acquired Pneumonia
SARI: severe acute respiratory infection
SNOMED CT: Systematized Nomenclature of Medicine Clinical Terms
WHO: World Health Organization
